# The effect of late presentation on HIV related mortality among adolescents in public hospitals of north showa zone Oromiya, Ethiopia; 2022: a retrospective cohort study

**DOI:** 10.1186/s12879-024-09550-3

**Published:** 2024-06-26

**Authors:** Misgana Kebede Gabre, Tadesse Bekele Tafesse, Leta Adugna Geleta, Cherugeta Kebede Asfaw, Henok Abebayehu Delelegn

**Affiliations:** 1 Department of Public Health, College of Health Sciences, Salale University, Fitche, Ethiopia; 2 Department of Pharmacy, College of Health Sciences, Salale University, Fitche, Ethiopia; 3 Department of Medicine, College of Health Sciences, Salale University, Fitche, Ethiopia

**Keywords:** Adolescence, Late presentation, HIV-related mortality, Ethiopia

## Abstract

**Background:**

Late human immunodeficiency virus (HIV) diagnosis is the most prominent cause of HIV/AIDS-related mortality and also increases the risk of transmission and spread of the disease in society. Adolescents are the most vulnerable population’s age group for HIV infection in several settings, but expanding access to early HIV testing remains a challenge. Consequently, a significant proportion of adolescents are still dying of HIV-related causes, and the current study aimed at assessing the effect of late presentation on HIV-related mortality among adolescents living with HIV.

**Methods:**

An institutional-based retrospective cohort study was conducted from August 21–November 21, 2022, at selected public hospitals in the North Showa Zone of Oromiya, Ethiopia. All adolescents living with HIV who had received no ART and presented for ART follow-up at public hospitals from September 1, 2012, to August 31, 2021, were included in the study. Data entry was done by Epi-data version 3.1.1 software and exported to Stata version 16 for further analysis. Both bi-variable and multivariable analyses were performed using the Cox proportional hazard model to compare the HIV-related mortality of early and late-presented adolescents using an adjusted hazard ratio at a 95% confidence interval (CI).

**Results:**

A total of 341 medical records of adolescents were included in the study, contributing an overall incidence rate of 3.15 (95% CI: 2.21–4.26) deaths per 100 person-years of observation throughout the total follow-up period of 1173.98 person-years. Adolescents with late presentation for HIV care had three times the higher hazard of mortality (adjusted hazard ratio (aHR) = 3.00; 95% CI: 1.22–7.37) as compared to those with early presentation for HIV/AIDS care. Adolescents within the age range of 15–19 years old (aHR = 3.56; 95% CI: 1.44–8.77), rural residence (aHR = 2.81; 95% CI: 1.39–5.68), poor adherence to ART (aHR = 3.17; 95% CI: 1.49–6.76), and being anemic (aHR = 3.09; 95% CI: 1.52–6.29) were other independent predictors of HIV-related mortality.

**Conclusion:**

The study found a substantial link between HIV late presentation to care and mortality among adolescents. Residence, age, antiretroviral therapy (ART) medication adherence, and anemia status were also found to be other independent predictors of HIV-related mortality. To achieve the ultimate aim of lowering mortality among adolescents living with HIV, rigorous emphasis must be placed on early presentation for HIV/AIDS care. In addition, counseling on adherence and prompt diagnosis and treatment of anemia are highly recommended to reduce mortality.

## Background

The human immunodeficiency virus (HIV) continues to be a major global health problem, and millions of people have died since it was originally discovered and isolated forty years ago. Worldwide, there were around 1.5 million new infections and 680,000 deaths from acquired immunodeficiency syndrome (AIDS) related causes in 2020 [1]. Adolescents (10 to 19 years of age) were the most severely affected age group by the epidemic, and they made up an increasing share of new HIV infections, accounting for about 5% of all people living with HIV and about 11% of new HIV infections among adults. Globally, in 2020, about 150,000 adolescents were newly infected and 32,000 died [2, 3]. HIV/AIDS is the second cause of mortality in adolescents worldwide, and the first in Africa [4, 5].

Late presentation to HIV care adversely affects the prognosis of AIDS by reducing the effectiveness of antiretroviral therapy (ART). It also increases the chance of transmission and spread of the disease, thereby increasing the risk of morbidity and mortality among the patients [6–8]. Studies revealed that some HIV-positive adolescents may die within the first year after starting [9, 10], and one of the prominent contributors to those deaths could be late presentation to HIV/AIDS care [11, 12]. In addition, adolescents are one of the unique age groups for whom HIV late presentation to care has a major impact on the outcome of the disease [13–15].

Despite the widespread availability of tests, expanding adolescent access to HIV testing (targeted and tailored testing strategies) continues to be a challenge, including index-linked testing, assisted partner notification, social network-based testing, and other novel modalities that boost the efficiency of testing programs [2]. As a result, adolescents become the age group most likely to be unaware of their diagnosis [16]. Some other unique contributing factors, like age of consent laws and low parent-adolescent communication about HIV/AIDS-related issues, can be barriers to adolescents accessing relevant HIV/AIDS services, resulting in late presentation to care and early mortality [17, 18].

In 2003, the Ethiopian government introduced its ART program and began implementing prevention, treatment, and care interventions to reduce HIV-related morbidity and mortality, improve the quality of life of patients living with HIV (PLWH), and mitigates some of the epidemic’s impacts. Also, drug and testing services are becoming more widely available in basic health care institutions and via campaign programs [19]. Even though in most developing countries like Ethiopia, where HIV testing and ART services are free, most adolescents present late for HIV/AIDS care and follow-up [13, 15]. The presence of some unique biological, physical, and social factors makes adolescents a more vulnerable age group in the population [16–18], and HIV-related deaths continue to be recorded in high numbers [7, 20]. Thus, designing and implementing high-impact, evidence-based interventions are necessary to reduce HIV-related mortality among adolescents. Therefore, this study aimed at determining the effect of HIV late presentation to care and other possible predictors on HIV-related mortality among adolescents attending ART clinics in public hospitals.

## Methods

### Study design and areas

A hospital-based retrospective cohort study was conducted from August 21–October 21, 2022, in selected public hospitals in the North Showa Zone of Oromiya region, Ethiopia. North Showa Zone has a total population of 1.6 million, with the capital city of Fitche located 114 km to the north of Addis Ababa. It is divided into 13 rural districts and two town administrations. There are 64 health centers and five public hospitals in the North Showa community that provide health care services. The study was carried out in three selected public hospitals, namely Salale University Comprehensive Specialized Hospital (SUCSH), Kuyu General Hospital, and Gundo-Meskel Primary Hospital.

### Study population and sampling

The source populations were all adolescents living with HIV who had received no ART and presented for ART follow-up at public hospitals in the North Showa Zone of Oromiya, Ethiopia. Adolescents who presented for ART follow-up from September 1, 2012, to August 31, 2021, at the three selected public hospitals were considered the study population and included in the study.

A standardized table of lists was prepared to extract eligible patients’ medical record numbers (MRNs) from the electronic databases of each health facility. The study included all patient charts that met the eligibility criteria. Patients’ charts that didn’t have full formal record information or had incomplete medical records for essential variables such as treatment outcome, age, and cluster of differentiation 4 (CD4) results, as well as those who didn’t have at least one documented clinic visit, were excluded from the study.

### Study variables

The study’s outcome variable was HIV/AIDS-related mortality. The primary exposure variable was the time of presentation to HIV care, which was categorized as early presentation versus late presentation for HIV care. Exposed patients are those with HIV late presentation to care (who present with a CD4 count below 350 cells/mm^3^), whereas non-exposed patients are those with early presentation for HIV/AIDS care (who present with a CD4 count ≥ 350 cells/mm^3^).

### Other independent variables

Socio-demographic variables such as age, sex, level of education and residence, past medical history of any opportunistic infections (OI) and clinical factors such as weight or body mass index (BMI), functional status, world health organization (WHO) clinical staging, and hemoglobin level.

### Data collection procedures and quality assurance

A data collection checklist was used to extract data from the patients’ charts. The tool was developed based on routine data registration protocols using standardized HIV care and follow-up forms (HIV/AIDS care monitoring and evaluation sheet) employed by ART clinics in health facilities in Ethiopia. Laboratory requests and ART intake forms were considered as a source of data and incorporated in the study. In each of the three selected hospitals, two data collectors were appointed to extract data from patients’ records, and another senior nurse supervised the data collection process. The patient’s chart was picked up from the chart room using MRN, and then data was extracted from the patient’s medical charts by using the prepared tool. During data collection, information that was not available on medical records was checked on smart care and other related registrations.

At Fiche number one health center, pre-testing was done with 5% of the total sample size. Based on the results of the pre-test, the check list was amended and modified. Training was given for the data collectors and supervisors on the entire data collection process. The supervisors checked the collected data on a daily basis for completeness and consistency.

### Data management and analysis procedures

Data entry was done by Epi-data version 3.1.1 software (Odense, Denmark) and then exported to Stata version 16 software for analysis. Prior to analysis, the data was cleaned for completeness using frequency and cross-tabulation. Recoding, categorization, and sorting of data were done using Stata software. Person-Month of follow-up was calculated by subtracting the date of HIV-related death (the last date of follow-up for censoring) from the date of HIV confirmation of patients.

During the analysis, descriptive statistics like frequency distribution, ratio, means, median, percentages, inter-quartile range (IQR), and standard deviation were calculated. Survival curves were used to further illustrate differences in hazard rations (HR), and with the Kaplan-Meier method, the survival probabilities of the exposed and unexposed groups were compared and interpreted accordingly. The log rank test was used to determine if the survival probabilities in the exposed and unexposed groups were significantly different. Both bivariable and multivariable Cox proportional hazard regression models were used to verify the effect of late presentation on HIV-related death of patients and other independent predictors of mortality, with an HR of 95% CI at 5% level of significance (α). Only variables with a *P*-value < 0.25 in the bivariable analysis were entered into the final model. The assumptions like proportionality of hazards over time, normality, and multi-collinearity were checked. The *P*-value of the proportionality of hazards over time in global test, using Schoenfeld residuals, was 0.914. Variables that were significant (*P*-value < 0.05) in the multivariable analysis were considered independent predictors of HIV-related mortality. Finally, the result was presented using texts, tables, figures, and diagrams.

### Operational definitions

#### Late presentation for HIV/AIDS care

defined as the enrollment of individuals for HIV/AIDS care with a CD4 count below 350 cells/mm^3^ [21].

#### Early presentation for HIV/AIDS care

defined as the enrollment of individuals for HIV/AIDS care with a CD4 count ≥ 350 cells/mm^3^.

#### HIV/AIDS related death or mortality

Defined as any recorded AIDS-related deaths other than deaths due to accidents like car accident, bullet injury, and any suicidal act while a patient is on ART and occurred in hospitals and any setting confirmed by the hospitals [22].

#### Censored patients

are those who had some information about individual survival time, but the exact time of presence of the outcome variable is unknown [23]. These individuals didn’t provide us the information whether HIV/AIDS related mortality had occurred or not. Adolescents who were alive beyond the end of follow-up period (August 31, 2021), lost/ drop from follow up, transferred out to other facilities, and if the cause of death was not HIV/AIDS related (accidental), considered as censored patients.

#### Functional status of patients

Classified according to the WHO criteria as working (W): capably to perform usual work in or out of the house, harvest, go to school or, for children, normal activities or playing; Ambulatory (A): capable to perform activities of daily living like self-care and going to the toilet unsupported; Bed-ridden (B): cannot perform activities of daily living like unable to go even to the toilet unsupported [24].

#### Anemia

In this study anemia is defined as anemic or not-anemic based on WHO criteria: i.e. Hemoglobin concentration *<* 12 g/dl for females and *<* 13 g/dl for males [25].

#### Nutritional status

Determined by the computed BMI and it shall be compared against a BMI- for- Age reference chart (BMI-for-Age Z-score), to decide whether the computed BMI indicates malnutrition or not; severely malnourished (< -3 SD), moderately malnourished (-2 to -3 SD), mildly malnourished (-1 to -2 SD) and normal weight (> -1 SD) [26].

## Results

### Socio-demographic characteristics of cohorts

The descriptive statistics extracted from the ART clinics of the three selected public hospitals revealed that the total number of adolescents on the ART cohort for the 10 years follow-up were 341 adolescents. After reviewing 341 medical records, 32 were excluded due to incompleteness, and 309 were included in the final analysis. The median age of participants was 17 years (IQR, 14–19 years); more than two-thirds, 220 (71.2%), of them were between 15 and 19 years of age. More than half, 181 (58.58%), of adolescents were females, and more than three-quarters, 209 (67.64%), lived in urban environments. Regarding participants’ occupations, 209 (67.64%) were students, and two-thirds, 186 (60.19%), were primary school students (Table 1).

**Table 1:** Socio-demographic characteristics of ALHIV followed from September 1, 2012 to August 31, 2021 at selected public hospitals in north Showa Zone of Oromiya, Ethiopia

Variables (*N* = 309)	Category	*N* (%)
Age classification [19]	10–14 years	89 (28.80)
(Median 17 years (IQR, 14–19 years))	15–19 years	220 (71.20)
Sex	Male	128 (41.42)
	Female	181 (58.58)
Level Of Education	No Formal Education	55 (17.8)
	Primary	186 (60.19)
	Secondary	56 (18.12)
	Tertiary	12 (3.88)
Place Of Residence	Urban	209 (67.64)
	Rural	100 (32.36)
Marital Status	Single	244 (78.96)
	Married	47 (15.21)
	Widowed	5 (1.62)
	Divorce	13 (4.21)
Client’s Occupation	Student	209 (67.64)
	Unemployed	59 (19.09)
	Employed	41 (13.27)

***Acronyms***: ALHIV; Adolescents Living with HIV, HIV; human immunodeficiency virus, IQR; Inter-Quartile Range

### Baseline clinical, laboratory and ART information

Regarding the baseline clinical and laboratory characteristics of adolescents living with HIV, 164 (53.07%) of them were late presented to HIV/AIDS care, and 145 (46.93%) were presented earlier. Most adolescents, 145 (46.93%), started ART after a week of being confirmed positive for HIV infection; 34 (11%) started within a week; and 130 (42.07%) started on the same day of confirmation.

At the time of enrollment, ALHIV had a median hemoglobin level of 12.9 mg/dl (IQR, 11.9–13.5 mg/dl); two-fifths, 124 (40.13%), were anemic. One hundred thirty-four (43.37%) adolescents were malnourished, and about 127 (41.1%) were given nutritional supplements at enrollment. Nearly half, 147 (47.57%), were categorized as having working functions, and about 6 (1.94%) adolescents were presented with edema. Regarding the patient’s tuberculosis (TB) co-infection status, 63 (20.39%) had a past TB treatment history, and nearly half of ALHIV, 150 (48.54%), had at least one OI (Table 2).

**Table 2:** Baseline clinical, laboratory and ART information; and incidence of death among ALHIV followed from September 1, 2012 to August 31, 2021 at selected public hospitals in north Showa Zone of Oromiya, Ethiopia

Variables (*N* = 309)	Category	*N* (%)
Baseline nutritional status (BMI Median = 17.8(IQR, 16.4–20.4 Kg/m^2^))	Normal nutritional status	175 (56.63)
	Mildly Mal-nourished	74 (23.95)
	Moderately Mal-nourished	46 (14.89)
	Severely Mal-nourished	14 (4.53)
Baseline anemia status (hgb level Median = 12.9(IQR, 11.9–13.5 mg/dl))	Non-anemic	185 (59.87)
	anemic	124 (40.13)
Clients’ functional status	Working	147 (47.57)
	Ambulatory	140 (45.31)
	Bedridden	22 (7.12)
Clients’ adherence level for ARV drug	Good ->95%	271 (87.7)
	Fair-85-94%	10 (3.24)
	Poor-<85%	28 (9.06)
Baseline Clients WHO stage	Stage I and II	148 (47.90)
	Stage III and IV	161 (52.10)
Past OI/Opportunistic cancer	No	159 (51.46)
	Yes	150 (48.54)
ARV drug regimen given at enrollment	1c	18 (5.83)
	4c	14 (4.53)
	1e	204 (66.02)
	1j	53 (17.15)
	others	20 (6.47)
Incidence of death	Censored	272 (88.03)
	Death	37 (11.97)

***Abbreviations/acronyms***:1c; Zidovudine + Lamivudine + Nevirapine, 4c; Zidovudine + Lamivudine + Nevirapine, 1e; Tenofovir Disoproxil Fumarate” + Lamivudine + Efavirenz, 1j; Tenofovir Disoproxil Fumarate + Lamivudine + Dolutegravir, CD4 cells; cluster of differentiation 4 cells, BMI; Body Mass Index, HIV; human immunodeficiency virus, ALHIV; Adolescents Living with HIV, ARV; Anti-ritro Viral, OI; opportunistic infection, WHO; World Health Organization, IQR; Inter-Quartile Range

More than half of the study participants, 161 (52.1%), were stage III or stage IV by WHO clinical staging. During the enrollment period, there were only 26 (8.41%) pregnant adolescents, and none of the others had received family planning services. More than four-fifths, 271 (87.7%), had good adherence levels for the ART drug in the first three months of follow-up, and more than two-thirds of adolescents, 204 (66.02%), were taking the 1e (tenofovir disoproxil fumarate” + lamivudine + efavirenz) ART drug regimen at the time of enrollment. Throughout the 10-year follow-up period, 37 (11.97%) adolescents were found to have died of direct HIV-related causes (Table 2).

### Incidence of HIV/AIDS-related mortality

From a total of 309 adolescents, two-thirds, 190 (61.49%), were active (alive on ART) at the end of the follow-up period, and 44 (14.24%) of them were transferred out to other health facilities. Throughout the 10-year follow-up period, about 25 (8.09%) adolescents were dropped from follow-up and 11 (3.56%) were lost. There were 2 (0.65%) participants who died due to other conditions. Out of the total 37 (11.97%) deaths directly related to HIV/AIDS causes, 31 (10.03%) adolescents died from late presentation to HIV/AIDS care, and 6 (1.94%) died from early presented to HIV/AIDS care.

**Fig. 1:**
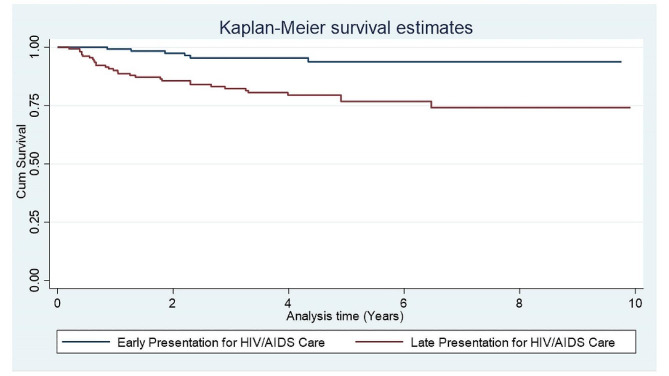
Kaplan–Meier survival estimate between early presented to HIV/AIDS care and lately presented to HIV/AIDS care among ALHIV followed from September 1, 2012 to August 31, 2021 at selected public hospitals in north Showa Zone of Oromiya, Ethiopia

The Kaplan–Meier survival plot from this study showed that the curve for unexposed group was consistently higher than that of exposed group. This indicated that early presented to HIV/AIDS care had a higher survival probability than those with lately presented throughout the ten years follow-up period. Moreover, as the number of years increases, the two curves appeared to get farther apart, suggesting that the beneficial effects of early presence to HIV/AIDS care over late presence are greater the longer one stays alive (Fig. 1). Survival of HIV patients within the ten years retrospective follow-up using Kaplan–Meier (the log rank) test for equality of survival function showed that there is a statistically significant difference between lately presented and early presented (*P*-value = 0.0001). The cumulative probability of surviving or being free from the event of interest at the end of 1, 4, 6 and 10 years was 94.4%, 86.6%, 84.7% and 83.2%, respectively.

The study participants contributed an overall 1173.98 person-years of follow-up. The person-year incidence of death was 1.08 per 100 adolescents-years in the unexposed group and 4.99 per 100 adolescents-years in the exposed group, with an overall incidence rate of 3.15 (95% CI: 2.21–4.26) per 100 adolescents-years. The attributable risk, which is the risk that is attributable only to the exposure group among the selected adolescents, is 3.92 per 100 adolescents-years of observation.

### Predictors of HIV-related mortality

Since there were multiple exposure variables, Hosmer and Lemeshow criteria for model development were utilized to find possible predictors of HIV-related mortality among adolescents. As a result, HIV late presentation to care in adolescents had a significantly higher hazard of HIV-related mortality with adjusted hazard ratio (aHR) = 3.00, 95% confidence interval (CI) 1.22–7.37, than early presentation to HIV/AIDS care (Table 3).

**Table 3:** Cox PH regression analyses model of baseline predictors for HIV/AIDS-related death among ALHIV followed from September 1, 2012 to August 31, 2021 at selected public hospitals in north Showa Zone of Oromiya, Ethiopia

Variables (*n* = 309)	Category	Patient Outcome		cHR (95% CI)	aHR (95% CI)
		Censored	Died		
		*N* (%)	*N* (%)		
Baseline CD_4_ count	≥ 350 cells/mm^3^	139 (95.9)	6 (4.1)	1	1
	< 350 cells/mm^3^	133 (81.1)	31 (18.9)	**4.71(1.96–11.29)**	**3.00(1.22–7.37)**
Age	10–14 years old	83 (93.3)	6 (6.7)	1	1
	15–19 years old	189 (85.9)	31(14.1)	**2.47(1.03–5.93)**	**3.56(1.44–8.77)**
Place of residence	Urban	196 (93.8)	13 (6.2)	1	1
	Rural	76 (76)	24 (24)	**4.44(2.26–8.73)**	**2.81(1.39–5.68)**
Adherence level for ARV drug	Good/fair	254 (90.4)	27 (9.6)	1	1
	Poor	18 (64.3)	10 (35.7)	**4.59(2.22–9.52)**	**3.17(1.49–6.76)**
Baseline anemia status	Non-anemic	172 (93)	13 (7)	1	1
	anemic	100 (80.6)	24 (19.4)	**2.86(1.45–5.62)**	**3.09(1.52–6.29)**
Baseline WHO Clinical staging	Stage I and II	143 (96.6)	5 (3.4)	1	NI
	Stage III and IV	129 (80.1)	32 (19.9)	**5.91(2.30-15.18)**	NI

Note: Data shown in bold indicates statistical significance (*P* < 0.05)

***Abbreviations/ acronyms***: aHR; Adjusted Hazard Ratio, CD4 cells; Cluster of Differentiation 4 cells, cHR; Crude Hazard Ratio, CI; Confidence Interval, HIV; Human Immunodeficiency Virus, ALHIV; Adolescents Living with HIV, NI; Not Included, WHO; World Health Organization

Adolescents within the age range of 15–19 years old had more than 3.5 times the higher hazard of death as compared to those within 10–14 years old (aHR = 3.56; 95% CI: 1.44–8.77). Rural residences had more than 2.8 times the higher hazard of mortality as compared to patients with urban residences (aHR = 2.81; 95% CI: 1.39–5.68). Patients with poor medication adherence had more than three times the higher hazard of death as compared to adolescents with good or fair adherence (aHR = 3.17; 95% CI: 1.49–6.76). ALHIV with a low baseline laboratory hemoglobin result (anemic) had a three times higher hazard of death as compared to adolescents with a normal baseline hemoglobin (non-anemic) status (aHR = 3.09; 95% CI: 1.52–6.29) (Table 3). Even though the baseline WHO clinical stage had significant statistics in the crude analysis, it was not included in the final model because it demonstrated collinearity with the main exposure variable (baseline CD4 cell count).

## Discussion

This research, using a multi-facility retrospective follow-up approach, is intended to investigate the influence of late presentation on HIV-related mortality among adolescents living with HIV in the selected public hospitals of North Showa Zone. The study found a significant association between late HIV presentation to care and mortality among ALHIV. It also indicated that place of residence; age, medication adherence, and anemia status were independent predictors of HIV-related death among ALHIV.

The overall incidence rate among ALHIV receiving ART was 3.15 per 100 person-years, with a total follow-up time of 1173.98 adolescent years. In this study, the mortality rate for ALHIV was lower than the rate identified in previous single-country studies, such as in India (4.9 deaths per 100 person-years) [27] and Zimbabwe (5.46 deaths per 100 person-years) [28]. The disparity may be attributed to variations in clinical features of research participants, as well as differences in study periods and different adolescents’ ages between studies (including children under the age of nine in their samples). However, the overall mortality rate in this study was higher than the rate reported by a global cohort collaboration across seven regions, with 0.97 deaths per 100 person-years [13]; in Ethiopia’s Amhara region, 1.52 deaths per 100 person-years [29]; and in another study done in Addis Ababa and the southern nations’, nationalities’, and peoples’ region (SNNPR), Ethiopia, 2.29 deaths per 100 person-years [19]. The difference in mortality rates between our study and previous studies, as well as the variation in mortality rates between these studies, could be attributed to sample sizes, categorizing ALHIV with an age of less than 15 years as an age proxy for perinatally acquired infection, and study settings, as this study included only hospitals.

Individuals with HIV late presentation to care had a three-fold higher hazard of death compared to adolescents with early presentation. Low CD4 counts at baseline were linked with an increased risk of death within one year after starting ART, and 16 adolescents died during the first year of follow-up. This is in line with the results of a worldwide cohort of collaborative research including adolescents living with HIV in Asia Pacific, the Caribbean, Central and South America, and Sub-Saharan Africa, which found that adolescents with low CD4 were more likely to die within a year of diagnosis [13]. Similar results were seen in numerous other studies from Haiti [30], Thailand [16], and Tanzania [9] which confirmed the present study’s conclusion that baseline CD4 cell count is a strong laboratory predictor of HIV-related mortality. In fact, CD4 cell count at ART initiation is the most significant predictor of disease development and dictates opportunistic infection (OI) risk categorization. It is also a direct indicator of a patient’s immunological state [8]. It is well known that a low CD4 count is a critical threshold for increased risk of death and is used similarly to the current WHO definition of advanced HIV disease [8]. In order to properly treat HIV/AIDS, infection should be detected as soon as feasible; when the person’s CD4 cell count exceeds 350 cells/mm^3^ [31]. This study’s findings also reinforce the previously stated fact by identifying HIV late presentation to care as a predictor of HIV-related mortality among ALHIV.

In this research, baseline blood hemoglobin levels were also a predictor of survival among HIV-positive adolescents. This conclusion was consistent with previous research from India and Ethiopia’s two regions, Addis Ababa and the SNNPR, which found that ALHIV with low baseline hemoglobin levels (clinically anemic) were more likely to die of HIV/AIDS than their counterparts [14, 27]. This could be explained by the fact that anemia at the baseline could be the result of HIV disease, particularly at an advanced stage, and it could also be a result of co-infection. Thus, HIV-infected patients with common hematologic complications have a higher mortality risk because anemic HIV-positive individuals take longer to recover from their disease or become completely unable to recover from their illness while on ARV drugs. Eventually, the illness’s horrible relationship with a significantly quicker rate of disease progression, poorer quality of life, and a strong prognostic marker for death [32, 33].

According to other studies, poor ART adherence in the first three months of enrollment may also contribute to adolescent mortality [29]. This might be explained by the fact that patients who do not comply with their therapy are in danger of developing medication resistance and, ultimately, treatment failure, which can lead to death if not caught early, since adherence is essential for suppressing viral replication and improving treatment success [34]. The importance of ART adherence in reducing ALHIV mortality and illness is a consistent finding [35].

Adolescents living in rural areas had a higher risk of death than urban patients and were found to be a significant predictor of HIV-related mortality. In keeping with current findings, several other studies have shown that rural residency is a demographic predictor of HIV survival in adolescents [15, 36]. The explanation for this similarity is that the research sites and study regions are comparable in resource-limited situations, where people living in rural areas have difficulty accessing health care information and services. In resource-limited settings, poor retention of adolescents throughout the HIV care cascade is also [30]. Being from a rural area may also be an indirect indication of poorer care quality in distant parts of the country [14].

In line with research undertaken in Sub-Saharan Africa (SSA) [37] this study found that being an older adolescent (15–19 years old) increased the risk of HIV/AIDS-related mortality when compared to younger adolescents (10–14 years old). The disparity in mortality rates between age groups may be due to evidence of differences in service delivery by age group; children under the age of 15 were more likely to enroll in designated pediatric care clinics, whereas youth over the age of 15 would have been enrolled and followed in the adult care clinic. It is probable that pediatric programs were better able to retain patients than adult programs [38, 39]. Furthermore, dealing with the potential of lifetime therapy is intimidating for older adolescents due to their typical maturation problems—emotionally, mentally, physically, and sexually [40].

The main limitation of this study is that, due to the retrospective nature of the research’s design, there were some missing data, some details of the patients’ clinical condition were underestimated, and there were incomplete records of variables or charts. Furthermore, the study’s small sample size may impair the precision of the results. Moreover, the study did not measure health-care quality, which may have an impact on HIV-related deaths.

## Conclusion

The study found a substantial link between HIV late presentation to care and mortality among adolescents living with HIV. HIV late presentation to care has increased mortality; hence, detecting HIV-infected adolescents as early as feasible might be one of the absolute answers to controlling death from HIV/AIDS. Rural residence, the age group of 15–19 years, poor medication adherence, and a low baseline hemoglobin level were also independent predictors of HIV-related death among ALHIV. To achieve the ultimate goal of lowering mortality among ALHIV, rigorous emphasis must be placed on ALHIV with poor baseline clinical and laboratory results, since their illness progression is faster and leads to death early. The study’s findings also highlight additional dimensions that future researchers should consider in large-scale prospective study designs.

## Data Availability

A supporting document (research ethics approval letter), that could be considered in the review and assessment of our manuscript, is provided within the related information file.

## Abbreviations

1c: Zidovudine + Lamivudine + Nevirapine
4c: Zidovudine + Lamivudine + Nevirapine
1e: Tenofovir Disoproxil Fumarate + Lamivudine + Efavirenz
1j: Tenofovir Disoproxil Fumarate + Lamivudine + Dolutegravir
AIDS: Acquired immunodeficiency syndrome
aHR: Adjusted Hazard Ratio
ALHIV: Adolescents Living with Human Immunodeficiency Virus
ART: Antiretroviral Therapy
BMI: Body Mass Index
CD4: Cluster of Differentiation 4
cHR: Crude Hazard Ratio
CPT: Cotrimoxazole preventive therapy
Hgb: Hemoglobin
HIV: Human Immunodeficiency Virus
HR: Hazard Ratio
INH: Isoniazid
IQR: Inter-Quartile Range
IRB: Institutional Review Board
LTFU: Lost to Follow-Up
MRN: Medical Record Number
OI: Opportunistic Infection
PMTCT: Prevention of Mother to Child Transmission
SSA: Sub-Saharan Africa
SUCSH: Salale University Comprehensive Specialized Hospital
TB: Tuberculosis
UNAIDS: United Nations Program on HIV/AIDS
UNICEF: United Nations International Children’s Emergency Fund
WHO: World Health Organization
